# Tales from the crypt and coral reef: the successes and challenges of identifying new herpesviruses using metagenomics

**DOI:** 10.3389/fmicb.2015.00188

**Published:** 2015-03-13

**Authors:** Charlotte J. Houldcroft, Judith Breuer

**Affiliations:** ^1^Infection, Inflammation and Rheumatology, Institute of Child Health, University College London, London, UK; ^2^Division of Infection and Immunity, University College London, London, UK

**Keywords:** metagenomics, virus discovery, next-generation sequencing, herpesviruses, *de novo* assembly

## Abstract

Herpesviruses are ubiquitous double-stranded DNA viruses infecting many animals, with the capacity to cause disease in both immunocompetent and immunocompromised hosts. Different herpesviruses have different cell tropisms, and have been detected in a diverse range of tissues and sample types. Metagenomics—encompassing viromics—analyses the nucleic acid of a tissue or other sample in an unbiased manner, making few or no prior assumptions about which viruses may be present in a sample. This approach has successfully discovered a number of novel herpesviruses. Furthermore, metagenomic analysis can identify herpesviruses with high degrees of sequence divergence from known herpesviruses and does not rely upon culturing large quantities of viral material. Metagenomics has had success in two areas of herpesvirus sequencing: firstly, the discovery of novel exogenous and endogenous herpesviruses in primates, bats and cnidarians; and secondly, in characterizing large areas of the genomes of herpesviruses previously only known from small fragments, revealing unexpected diversity. This review will discuss the successes and challenges of using metagenomics to identify novel herpesviruses, and future directions within the field.

## Introduction

Herpesviruses are double-stranded DNA viruses which infect many members of the animal kingdom, including mammals, birds and reptiles (McGeoch and Gatherer, 2005), fish (Lepa and Siwicki, 2012), amphibians (Davison et al., 2006), molluscs (Nicolas et al., 1992; Savin et al., 2010), and cnidarians (Vega Thurber et al., 2008; Grasis et al., 2014). They typically infect a large proportion of their target population, spreading through a variety of horizontal and vertical routes. They can cause disease in many settings, e.g., hemorrhage disease in elephants caused by elephant endothelial herpesviruses (Wilkie et al., 2014); cancers caused by the human gammaherpesviruses Epstein-Barr virus (EBV) and Kaposi’s sarcoma-associated herpesvirus (KSHV) (Taylor and Blackbourn, 2011); pulmonary infection in cats caused by feline herpesvirus 1 (Thiry et al., 2009) and terrapene herpesvirus 1-associated pneumonia in Eastern box turtles (Sim et al., 2014); seasonal mass mortality in oysters (Nicolas et al., 1992); and herpes simplex virus (HSV) encephalitis in humans (Whitley, 2006). Disease may be associated with primary infection, reactivation of latent infection, immune suppression, or immune senescence. Previous approaches to herpesvirus discovery have utilized a range of methods, discussed in greater depth elsewhere (Bexfield and Kellam, 2011). Briefly, these include: electron microscopy [EBV and cytomegalovirus (CMV); Ho, 2008], PCR-based representational difference analysis (KSHV; Chang et al., 1994), DNA *in situ* hybridization (chelonid herpesviruses Teifke et al., 2000), immunohistochemistry (alcelaphine herpesviruses Klieforth et al., 2002), and polymerase chain reaction (PCR) and Sanger sequencing (primate rhadinoviruses Lacoste et al., 2001, and lizard herpesviruses Wellehan et al., 2004; Literak et al., 2010). Metagenomics is the newest approach to add to this list: a deep-sequencing-based analysis of the nucleic acid contents of a cellular or acellular sample, unreliant on tissue culture and without reference to prior knowledge of which viruses are present in a sample.

Because herpesviruses are widespread among animals, herpesvirus-like sequences are likely to be present in many metagenomic studies. There are also features of herpesvirus molecular biology which increase the likelihood that deep-sequencing studies which are not explicitly metagenomic in nature will nevertheless detect herpesviruses, which may or may not be disease-associated. As widespread DNA viruses, any animal genome sequencing project that uses DNA from primary tissue or samples (e.g., saliva or blood) is likely to include sequences from herpesviruses present in that organism. Even though virus detection is not a primary target of host genome sequencing studies, viruses present in the sample will form a proportion of the sequence reads, and herpesviruses have been discovered in exactly such data (Aswad and Katzourakis, 2014). In this review article, we summarize some of the success stories in identifying novel herpesviruses through metagenomics, and offer a warning on the topic of virus discovery.

## Finding the Needle in a Haystack

Much herpesvirus genome sequencing in the past has relied upon large volumes of DNA, acquired through culturing the virus in permissive cell lines (Baer et al., 1984; Lei et al., 2013; Lin et al., 2013). In uncultured or unenriched samples, herpesvirus DNA is present in much smaller proportions than host DNA (Depledge et al., 2011). Herpesvirus genomes are typically hundreds of kilobases in length, complicating the task of Sanger or ultra-deep sequencing of herpesvirus genomes. The problem of poor virus to host DNA ratios applies equally to herpesvirus discovery. It is perhaps unsurprising that many herpesviruses are identified using consensus or degenerate PCR primer sets, but their genomes remain unsequenced (e.g., Bodewes et al., 2014; Sim et al., 2014). The success of identifying novel herpesviruses or of characterizing their genomes further is influenced by the similarity of the novel herpesvirus to related viruses. Some uncharacterized herpesviruses may map well to related herpesviruses, and are relatively easy to assemble from deep-sequence data, or design consensus primers to amplify. Divergent herpesviruses are more difficult to detect and assemble using traditional methods, mapping poorly to existing reference sequences (Pop, 2009). Highly similar and divergent herpesviruses alike may require *de novo* assembly to generate accurate consensus sequences, and potentially further PCR and sequencing to fill gaps in the assembly or confirm novel sequences (e.g., Babra et al., 2012). Metagenomics has an important role in identifying and characterizing divergent herpesviruses, and in allowing the discovery of herpesviruses in hosts or tissue types they might not be expected to be found in.

## Deep-Sequencing of Deltaherpesviruses

Elephant endotheliotropic herpesviruses 1A (EEHV1A) and 1B (EEHV1B), causes of hemorrhage disease in African and Asian elephants, required deep sequencing and *de novo* assembly to characterize their complete genome sequences (Wilkie et al., 2013). EEHVs cannot yet be propagated in tissue culture and must be sequenced from primary tissue—in this case, necropsy tissue from a juvenile Asian elephant. While EEHVs were known of from traditional PCR and Sanger-based methods, the complete genome sequence proved to be divergent from known herpesviruses, with 60 novel herpesvirus genes identified in the EEHV1A genome (Ling et al., 2013). Whole genome sequencing of EEHV5 revealed further genetic diversity between strains, with only 60% similarity between EEHV1A and EEHV5 (Wilkie et al., 2014), and 25% nucleotide divergence between coding regions of EEHV1A and EEHV2 (Richman et al., 2014). The use of deep-sequencing to resolve the genome sequences of EEHVs expands our knowledge of herpesvirus diversity, and may lead to the identification of putative deltaherpesviruses in other species.

## Herpesviruses Hiding in Plain Sight

Public sequence repositories containing mapped and unmapped reads from large genome-sequencing projects are fertile territory for identifying novel herpesviruses. Untargeted deep and ultra-deep sequencing of mammalian genomes from primary samples can serve as metagenomic datasets for researchers able to re-analyze the data in ways that may not have been anticipated in the original project. Aswad and Katzourakis (2014) screened 14 whole genomes from nine primate sequences available in GenBank for known herpesvirus protein sequence clusters, hoping to identify novel herpesviruses. Characterizing primate herpesvirus diversity is of interest to researchers for a number of reasons: herpesviruses have both zoonotic (Hummeler et al., 1959) and anthroponotic (Huemer et al., 2002) potential in primates, with high fatality rates for humans and non-human primates (Estep et al., 2010); increasing our knowledge of herpesviruses in other species may provide new model systems for understanding how herpesviruses cause disease in humans (Staheli et al., 2014); and the identification of putative endogenous herpesviral elements within primate genomes serves as a fossil record for the herpesviridae (Aswad and Katzourakis, 2014).

Aswad and Katzourakis successfully identified novel herpesvirus-like sequences, and constructed partial herpesvirus genomes, from the whole genome sequences of two primates not previously known to carry herpesviruses: the aye-aye (*Daubentonia madagascariensis*) and Philippine tarsier (*Tarsius syrichta*). Aswad and Katzourakis analyzed both mapped and unmapped reads from the primate genomes, enabling them to identify both exogenous, and chromosomally integrated, potentially endogenised, herpesviruses. The tarsier herpesvirus sequences were particularly interesting because they are the first report of a potentially endogenous herpesvirus, related to human herpesvirus 6B which chromosomally integrates in a small proportion of humans (Luppi et al., 1993; Tanaka-Taya et al., 2004). The tarsier herpesvirus sequences they reported were heavily mutated, not replication competent and contiguous with stretches of host DNA, supporting the view that this virus has become fully endogenised in tarsier evolutionary history. They were also able to assemble much larger regions of the bonobo (*Pan paniscus*) herpesvirus *Pan paniscus lymphocryptovirus 1* (PpanLCV1), covering an estimated 45% of its genome (∼78,000 bp), which was only previously known from small fragments (∼3000 bp; Ehlers et al., 2003).

While a successful strategy for discovering novel herpesviruses, this approach differs from that employed by metagenomics studies. The authors acted with a reasonable assumption that herpesviruses might be present in the data they were screening, searching only for sequences with similarity to previously reported herpesvirus-like sequences.

Successful identification of novel herpesviruses in host genome sequence datasets also relies on the genomes being sequenced from primary tissue (blood and liver biopsy, in two cases) rather than cell lines, which do not contain the diversity of viruses found in a primary tissue sample. For example, the bonobo genome was sequenced from a bonobo lymphoblastoid cell line which was immortalized in the laboratory using the human herpesvirus EBV (Prufer et al., 2012); this dataset is therefore much more likely to contain human EBV sequences, present in every cell, than primate gammaherpesviruses. Utilizing uncultured primary samples is at the heart of metagenomics.

## Going Batty for Metagenomics

Metagenomics may be seen as the opposite side of the coin to host genome sequencing projects: while host genome sequence assembly discards all sequences which are not on target to the host genome, metagenomic studies focus on non-host sequences (reviewed in Mokili et al., 2012). Eliminating or reducing the amount of host nucleic acid present in sequencing data sets has been addressed in some metagenomics studies of bat viral diversity, with success—three recent studies of chiropteran (bat) metagenomes have identified novel herpesvirus sequences, findings confirmed by a fourth study. Bats represent a fifth of the classified mammalian species on earth, and are thought to be a significant reservoir of emerging infectious diseases, particularly those bats found in urban areas or used by humans as a food source (Luis et al., 2013).

Straw-colored fruit bats (*Eidolon helvum*) are widely distributed across Africa and eaten as bushmeat—and therefore a potential zoonotic reservoir (Baker et al., 2013). Studying the viral diversity of this bat is therefore of significant interest to researchers. The sampling approach taken by Baker et al. (2013) was to pool the nucleic acids taken from three sample types: urine, collected non-invasively, and throat and lung swabs obtained from wild, anaesthetized bats from rural and urban locations in Ghana, with sequences assembled *de novo*. They identified sequences from previously unknown bat herpesviruses, with most reads belonging to beta and gammaherpesviruses. The largest proportion of herpesviral reads were obtained from throat swabs, underlining the importance of choosing appropriate primary samples in herpesvirus discovery. The presence of novel herpesviruses was then confirmed by PCR amplification of fragments from the original samples.

Multiple novel herpesviruses were also discovered in a sample of bats from China, which included a broader sampling strategy than in the Baker et al. (2013) study. Anal and pharyngeal swaps were collected from 11 bat species (Wu et al., 2012). In order to maximize the ratio of viral to host/eukaryotic nucleic acid, samples were passed through a filter to remove bacterial and eukaryotic cells. Sequence-independent nucleic acid amplification was used to increase the available genetic material for sequencing, and deep-sequence reads were aligned to the NCBI nt database, identifying novel bat herpesviruses. Even after physical filtering, only 1.2% of sequencing reads were viral, but among these reads, they were able to identify sequences from two previously unknown betaherpesviruses and two gammaherpesviruses. Wu et al. (2012) then used PCR amplification and genome walking to confirm the presence of novel herpesviruses and obtain longer sequences for phylogenetic analysis from the original sample. Clearly low herpesvirus abundance in the initial sample was a factor in preventing whole-genome assembly from the primary material; but metagenomics was integral to identifying the viruses.

A similar study of seven bat species from eastern North American detected a further novel bat betaherpesvirus (Donaldson et al., 2010), from saliva and fresh feces. Samples were passed through a filter to reduce non-viral nucleic acid carry-over, and pooled by species, sex, and age for sequencing to increase the total pool of nucleic acid available. Sequence-independent sample amplification with random hexamers was used, followed by deep-sequencing and *de novo* assembly.

A metagenomic study of French bats (Dacheux et al., 2014) which examined lung, liver and brain tissue from the carcasses of five species was also able to find evidence of the herpesviruses reported by previous studies, reinforcing the ubiquity of herpesviruses among the chiropterans. It is further evident from these studies that saliva, throat swabs and primary tissue samples are integral to herpesvirus discovery.

## An Ocean of Herpesviruses

Metagenomics is changing our ideas of where future herpesviruses may be discovered, providing evidence of herpesvirus-like DNA in cnidarians. A metagenomic study of a salt-water coral virome revealed herpesvirus-like sequences in *Porites compressa*. The researchers performed deep-sequencing of the coral in optimal and “stressed” conditions, including increased temperatures, increased acidity, and nutritional stress. They found a small number of herpesvirus-like reads in the coral harvested under optimal conditions, but a much greater number of herpesvirus-like reads were identified in the stressed coral samples. The researchers suggest this is due to lytic reactivation of herpesviruses within the coral under conditions of stress, reflecting what is known about herpesvirus reactivation in mammals. They were able to PCR amplify and sequence a gene with moderate identity to the thymidylate synthase gene from Herpesvirus saimiri 2 (Vega Thurber et al., 2008).

The same research group followed this up with a metagenomic analysis of four species of coral (*Acropora*, *Diploria*, *Montastraea*, and *Porites*) to test for associations between herpesvirus-like sequence abundance and disease in cnidarians (Soffer et al., 2014), and used transmission electron microscopy to identify herpesvirus-like particles within the cells of healthy coral. Using *de novo* assembly, they found that herpesvirus-like sequences were more common in healthy coral than diseased coral. This result contradicts the previous study, and highlights the difficulties in making quantitative comparisons between metagenomic datasets, when sequences from novel viruses cannot be normalized to gene or genome length for quantification in the manner of RNA-seq data (Mortazavi et al., 2008). While herpesvirus-like sequences had previously been detected in molluscs (e.g., Davison et al., 2005), metagenomics was instrumental in finding herpesvirus-like sequences in cnidarians. Subsequent metagenomic studies of fresh water cnidarians have also found herpesvirus-like sequences in a number of species of hydra (Grasis et al., 2014).

## Novel Virus or Novel Contaminant?

There are warnings attached to highly sensitive deep-sequencing and metagenomic discovery of any virus: principally, the risk of contamination. Deep-sequencing and *de novo* assembly of viral nucleic acids provide unprecedented opportunities to identify new viruses, some of which may be pathogenic, but these technologies may also detect contaminating nucleic acids that can be introduced to the sample extraction and sequencing pipeline at many different points.

There have been a number of high profile reports over the last 5 years of “novel” viruses (Xu et al., 2013), which have later been identified as nucleic acid contaminants from commercial sequencing products (e.g., Naccache et al., 2013, 2014; Rosseel et al., 2014). The origins of these nucleic acids are as diverse as mice (Erlwein et al., 2011) and algae (Naccache et al., 2013, 2014). Viral contamination of cell lines (Hue et al., 2010), especially those which have been xenografted during their life in tissue culture (Griffiths et al., 1997, 2002; Paprotka et al., 2011), has also proven to be a significant problem. The story of these “rumor viruses” (Weiss, 2010) must serve as a constant reminder to the virus discovery and metagenomics community. Furthermore, the problem is not confined to viruses, encompassing bacterial contamination of nucleic acid extraction spin columns (Salter et al., 2014) and other laboratory reagents. Screening of control samples and potentially of common laboratory reagents used as each point of the sample preparation process may be necessary for researchers to be confident that they have identified a novel herpesvirus.

The need for further biological characterization to clarify the relationship between a novel pathogen and a disease is a well-recognized problem in viruses studied primarily from molecular data (reviewed in Lipkin, 2010). For example, Bodewes et al. (2014) explicitly comment on the problem of establishing etiology in their report of a novel herpesvirus in harbor and gray seals, initially discovered in juvenile seals with ulcerative gingivitis but also found to be present in a large proportion of healthy controls.

## Methodology Influences Success

Metagenomic and deep-sequence analysis of a wide range of eukaryotic organisms over the last decade has shown that herpesviruses are more diverse and ubiquitous than previously imagined. Successful identification of novel herpesviruses or related viruses will rely on the availability of high-quality DNA and RNA from suitable primary samples.

While samples collected non-invasively, such as feces or urine, are immediately appealing for virus discovery because they are easy to collect, they may not be the ideal source material for identifying novel herpesviruses. Metagenomic analysis of bats found most herpesvirus-related sequence reads in throat swab or saliva samples. The primate sequence data in which novel herpesviruses were identified in public sequence repositories were initially sampled from blood and liver. There is an obvious correlation with the cellular tropism of individual herpesvirus species, their abundance in a particular tissue, and the likelihood of discovering a herpesvirus. Herpesviruses with a tropism for lymphocytes may be most readily detectable in blood samples or lymph node biopsies, for example. The physiological and environmental stresses affecting the host may also be an important variable in novel herpesvirus detection.

Metagenomics is an important tool in characterizing herpesviruses previously only known of from sequencing of short PCR amplicons. The success of metagenomics methods improves as the proportion of viral reads within a sample increases. Enriching for total viral material within a sample can be achieved by physical methods early in the sample preparation process, physically reducing the amount of contaminating non-viral nucleic acid using combinations of centrifugation, filtration and nuclease treatments (reviewed in Hall et al., 2014). Further enrichment is then possible, either sequence-independent enrichment using random hexamers or targeted enrichment (such as Sure Select) of specific viruses. All of these approaches may be needed to assemble whole genomes of herpesviruses from uncultured primary samples (Depledge et al., 2011). Targeted enrichment of herpesviruses relies on whole genome sequences being available for the virus of interest. Deep sequencing data from metagenomic studies can provide the sequences needed to design an initial set of PCR primers, as with phocine herpesvirus 7 (Bodewes et al., 2014), or target enrichment baits so that further sequence information can be collected. Metagenomics has also identified novel viruses from many other families, including coronaviruses (Schurch et al., 2014), papillomaviruses (Canuti et al., 2014), and tornoviruses (Ng et al., 2009), to give just a few examples.

Our closest living relatives, chimpanzees, bonobos and gorillas, are a rich source of human herpesvirus-like agents; whole-genome sequences of these viruses would tell us about our shared evolution with these ancient and successful pathogens. The discovery of two previously unknown primate herpesviruses in public sequence repositories (Aswad and Katzourakis, 2014) makes clear the possibilities for identifying further novel primate herpesviruses through metagenomics in the near future.

There is also the possibility of discovering further human herpesviruses. Humans are unusual among great apes because they have only one rhadinovirus species (KSHV), not two. The presence of three highly divergent forms of KSHV gene K15, including a form found only in southern Africa, has been suggested by some authors (Hayward and Zong, 2007) to indicate that there may be (or may have been) an unknown second KSHV-like rhadinovirus in humans. Deep sequencing and metagenomic analysis of primary tissue samples has been successful in identifying other primate herpesviruses, and as our knowledge of herpesvirus sequence diversity increases, additional human herpesviruses may be identified.

Metagenomics is a promising technique that provides a further tool to identify novel herpesviruses, reducing our reliance on PCR for herpesvirus discovery while also improving our ability to design PCR primers or baits for sequencing studies. Furthermore, metagenomics is increasing our knowledge of the diversity and evolutionary history of herpesviruses; and emphasizing the importance of studying primary samples rather than cell lines or cultured virus isolates if we wish to discover further novel herpesviruses.

### Conflict of Interest Statement

The authors declare that the research was conducted in the absence of any commercial or financial relationships that could be construed as a potential conflict of interest.

## Abbreviations

EEHV: elephant endotheliotropic herpesviruses
LCV: lymphocryptovirus.
